# Geranylgeranyl isoprenoids and hepatic Rap1a regulate basal and statin-induced expression of PCSK9

**DOI:** 10.1016/j.jlr.2024.100515

**Published:** 2024-02-02

**Authors:** Yating Wang, Brea Tinsley, Stefano Spolitu, John A. Zadroga, Heena Agarwal, Amesh K. Sarecha, Lale Ozcan

**Affiliations:** 1 Department of Medicine, Columbia University Irving Medical Center, New York, NY, USA; 2 Department of Cardiology, The Second Xiangya Hospital, Central South University, Changsha, China

**Keywords:** PCSK9, LDL, Rap1a, statins, geranylgeranylation

## Abstract

LDL-C lowering is the main goal of atherosclerotic cardiovascular disease prevention, and proprotein convertase subtilisin/kexin type 9 (PCSK9) inhibition is now a validated therapeutic strategy that lowers serum LDL-C and reduces coronary events. Ironically, the most widely used medicine to lower cholesterol, statins, has been shown to increase circulating PCSK9 levels, which limits their efficacy. Here, we show that geranylgeranyl isoprenoids and hepatic Rap1a regulate both basal and statin-induced expression of PCSK9 and contribute to LDL-C homeostasis. Rap1a prenylation and activity is inhibited upon statin treatment, and statin-mediated PCSK9 induction is dependent on geranylgeranyl synthesis and hepatic Rap1a. Accordingly, treatment of mice with a small-molecule activator of Rap1a lowered PCSK9 protein and plasma cholesterol and inhibited statin-mediated PCSK9 induction in hepatocytes. The mechanism involves inhibition of the downstream RhoA-ROCK pathway and regulation of PCSK9 at the post-transcriptional level. These data further identify Rap1a as a novel regulator of PCSK9 protein and show that blocking Rap1a prenylation through lowering geranylgeranyl levels contributes to statin-mediated induction of PCSK9.

Lipid-lowering therapies remain the cornerstone treatment of atherosclerosis, and continual improvements in therapies are still at the forefront of discovery. Proprotein convertase subtilisin/kexin type 9 (PCSK9) inhibitors emerged as a novel treatment approach to lower serum LDL-C and are well tolerated (1, 2). PCSK9 binds to LDL receptor (LDLR) and increases its lysosomal degradation, which results in decreased rate of LDL-C removal from the circulation (3). PCSK9 may also contribute to atherosclerosis development through its effects on endothelial and vascular smooth muscle cells (4, 5). Accumulating evidence suggest that PCSK9 inhibition has many beneficial effects besides LDL-C management, including lowering of atherogenic Lp(a) levels and protecting against calcific aortic valve stenosis (6, 7, 8). Anti-PCSK9 antibodies lower LDL-C and reduce coronary events; however, their high costs and low patient compliance limit their use (9). Liver-targeted siRNA against PCSK9 also robustly lowers LDL-C, but unlike the well-tolerated genetic variants, it removes all PCSK9 from the cell, raising safety concerns (10). Thus, further studies aimed at identifying the mechanism(s) of PCSK9 regulation are crucial, which could be used to develop new therapeutic strategies.

Statins inhibit HMG-CoA reductase, which catalyzes the rate-limiting step in cholesterol synthesis, and upregulate LDLR (11). Although statins are the most widely prescribed drugs to lower cholesterol, a substantial proportion of patients do not reach target LDL-C goals despite receiving maximally tolerated statin therapy (12, 13, 14, 15). In fact, each doubling of the statin dose was shown to produce an average additional decrease in LDL-C levels of about only 6% (rule of 6) (16, 17). This was attributed in part to an increase in PCSK9 levels upon statin use, which promotes LDLR degradation and decreases statins’ LDL-C-lowering efficacy (18, 19, 20, 21, 22). Accordingly, combination of anti-PCSK9 antibodies with statins provides 50–60% additional reduction in LDL-C and reduces cardiovascular events by 50% (23). On the other hand, clinical trials of selective cholesterol absorption inhibitor, ezetimibe, have not shown an effect on plasma PCSK9 levels, suggesting that PCSK9 induction by statins may not be simply attributed to their effects on plasma LDL-C (20, 24). In this regard, there is a need to understand the underlying molecular mechanisms of this ironic relationship between statins and plasma PCSK9 that can lay a path to improve their efficacy. An increase in nuclear localization of SREBP2, which binds to the SRE motifs located in the promoter region of *Pcsk9* gene, has been shown as one mechanism by which statins adversely increase PCSK9 levels (25, 26). It is unknown, however, whether there are additional pathways involved in statin-mediated PCSK9 induction. In addition to lowering cholesterol synthesis and regulating LDL-C levels, statins also inhibit the formation of other mevalonate pathway metabolites, including farnesyl pyrophosphate and geranylgeranyl pyrophosphate (GGPP) isoprenoids, which are necessary for the prenylation and activation of small G proteins, including Rap1a (27, 28, 29).

Rap1a is a small GTPase, which gets activated by GTP loading (30, 31, 32, 33). Attachment of GGPP isoprenoid chain to Rap1a by geranylgeranyl transferase-I (GGTase-I) results in Rap1a′s membrane localization and its activation by guanine exchange factors, including exchange factor activated by cAMP-2 (Epac2) (34, 35). The GTPase-activating protein, Rap1GAP, hydrolyzes Rap1a′s GTP and inactivates it. Our previous work revealed that activation of glucagon receptor-cAMP-Rap1a pathway in the liver decreases hepatic and plasma PCSK9, which leads to increased LDLR protein and lower plasma LDL-C (36). Specifically, we reported that silencing of Rap1a in isolated hepatocytes increases PCSK9 and acute inhibition of Rap1a activity in mice liver via adenovirus-mediated overexpression of Rap1GAP elevates plasma PCSK9, lowers hepatic LDLR, and increases LDL-C. Our mechanistic studies revealed that Rap1a decreases the stability of PCSK9 protein without changing its mRNA levels or SREBP2 activity (36). In addition to its LDL-C-lowering effect, we recently showed that activation of Rap1a suppresses hepatic glucose production, and its inhibition contributes to obesity-induced glucose intolerance (37). Our work further revealed that statins decrease Rap1a prenylation and activity in isolated hepatocytes and human liver samples, and this may in turn explain their hyperglycemic effects (37).

Because Rap1a activity is decreased upon statins and adding back geranylgeranyl isoprenoids restores hepatic Rap1a activity, we became interested in the role of GGPP and Rap1a in statin-mediated PCSK9 induction (37). In this study, we show that restoration of GGPP levels in statin-treated mice lowers statin-induced PCSK9 and further decreases plasma LDL-C, independent of a change in *Pcsk9* mRNA. Moreover, treatment of mice with a small-molecule activator of Rap1a recapitulates the Rap1a gain-of-function model and lowers plasma PCSK9 and cholesterol levels. Rap1a activator treatment also lowers statin-induced PCSK9 in hepatocytes. We further report that Rap1a regulates PCSK9 via inhibiting the RhoA-ROCK pathway. These findings provide an additional mechanistic insight into the paradoxical regulation of PCSK9 by statins and reveal that GGPP-Rap1a pathway contributes to the post-transcriptional regulation of PCSK9.

## Materials and Methods

### Mouse experiments

WT (stock #000664) and diet-induced obese (DIO, stock #380050) mice were from Jackson Labs. DIO mice were fed a high-fat diet with 60% kcal from fat (Research Diets). To avoid the effects of gender-related differences in metabolism, male mice were used for most of the experiments. All mice were maintained on a 12 h light-dark cycle. Recombinant adeno-associated viruses (AAVs, 1–2 × 10^9^ genome copies per mouse) and adenoviruses (0.75 × 10^9^ plaque-forming units per mouse) were delivered to DIO mice by tail vein injections, and experiments were commenced after 3–28 days. In some experiments, high cholesterol-containing diet fed WT mice were used, as indicated in the corresponding legends (38). For some of the statin treatment experiments, 18-week-old male DIO mice were treated orally with rosuvastatin (10 mg/kg/day) for 3 weeks, or 0.02% (w/w) simvastatin was mixed with the high-fat diet. Geranylgeraniol (GGOH) was given by oral gavage at a dose of 50 mg/kg/day for 3 weeks. About 20-week-old male DIO mice were treated intraperitoneally with 8-pCPT (8-pCPT-2′-O-Me-cAMP) 1.5 mg/kg/day for 2 weeks. For all experiments, male mice of the same age and similar weight were randomly assigned to experimental and control groups. All animal experiments were performed under the protocols, which were approved by the Columbia University Irving Medical Center Animal Care and Use Committee.

### Reagents and antibodies

Rosuvastatin (catalog no.: 18813), simvastatin for cell culture (catalog no.: 10010344), GGPP (catalog no.: 63330), specific Epac activator, 8-pCPT (catalog no.: 17143) and GGOH (catalog no.: 13272) were from Cayman Chemicals. C3 (Rho inhibitor I, catalog no.: CT04) was from Cytoskeleton. Anti-β-actin (catalog no.: 4970), anti-Hsp90 (catalog no.: 4877), anti-RhoA (catalog no.: 21117), anti-Gapdh (catalog no.: 5174), and anti-pan-Cadherin (catalog no.: 4068) antibodies were from Cell Signaling Technology. Anti-PCSK9 antibody was from R&D Systems (catalog no.: AF3985). AAV8-shRNA targeting murine *Pggt1b* was made by annealing complementary oligonucleotides 5′-CACCAAAGCCATCAGCTACATTAGAAGAAGTCAAGAGCTTCTTCTAATGTAGCTGATGGCTT -3′, which were then ligated into the self-complementary AAV8-RSV-GFP-H1 vector as described previously (39). The resultant constructs were amplified by Salk Institute Gene Transfer, Targeting, and Therapeutics Core. AAV8-sh-RhoA was as described before (38). siRNA sequences against murine *Rap1a*, *Pggt1b*, *Rhoa*, and *Rock* were purchased from Integrated DNA Technologies. Adenoviruses encoding LacZ and Rap1Gap were described previously (31, 40) and amplified and purified by Viraquest, Inc (North Liberty, IA). Myc-tagged WT Rap1a or myc-tagged prenylation deficient Rap1a (SAAX-Rap1a) plasmids were gifts from Dr Carol L. Williams (Medical College of Wisconsin) (41).

### Measurement of cholesterol and PCSK9

Plasma total cholesterol levels were measured using a colorimetric kit (Wako). Plasma lipoproteins were separated by KBr density ultracentrifugation as described previously (42). Briefly, equal amounts of mouse plasma (50–100 μl) were used for sequential density ultracentrifugation to separate VLDL (d < 1.006 g/ml), LDL (d = 1.006–1.063 g/ml), and high-density lipoprotein (d = 1.063–1.21 g/ml) in a TLA 100 rotor, and LDL fractions were used to measure cholesterol levels using the above colorimetric kit. Plasma PCSK9 levels were analyzed using an ELISA kit (R&D Systems).

### Hepatocyte experiments

Primary mouse hepatocytes were isolated from 12- to 20-week-old male or female WT mice as described previously (43). In some experiments, primary mouse hepatocytes were isolated from hepatocyte-specific Rap1a-deficient mice that were obtained by injecting *Rap1a*^fl/fl^ mice (Jackson Labs, stock number: 021066) with AAV-expressing Cre recombinase, driven by the thyroxin-binding globulin (TBG) promoter (AAV8-TBG-Cre). Hepatocytes were transfected with plasmids 10–12 h after plating, and experiments were conducted 48–72 h later. Transfections with scrambled, si-Rap1a, si-Pggt1b, si-RhoA, or si-ROCK constructs were carried out using Lipofectamine RNAiMAX reagent according to the manufacturer's instructions.

### Immunoblotting

Liver protein was extracted using RIPA buffer (Thermo Scientific), and the protein concentration was measured by DC protein assay kit (Bio-Rad). Whole-cell lysates were harvested in 2× Laemmli buffer. Cell extracts were electrophoresed on SDS-polyacrylamide gels and transferred to 0.2 μm or 0.45 μm PVDF membranes. Blots were blocked in Tris-buffered saline with 0.1% Tween-20 containing 5% BSA at room temperature for 1 h. Membranes were then incubated overnight at 4^o^C with primary antibodies. The protein bands were detected with horseradish peroxidase-conjugated secondary antibodies (Jackson ImmunoResearch) and Supersignal West Pico enhanced chemiluminescent solution (Thermo).

### Rap1a activity assay

Rap1a activity was assayed using Active Rap1 Detection Kit (Cell Signaling Technology, catalog no.: 8818). Liver tissue sample was lysed with lysis/binding/washing buffer containing 2 mM PMSF, 5 μg/ml leupeptin, 10 nM okadaic acid, and 5 μg/ml aprotinin. GST-RalGDS-RBD fusion protein was used to bind the activated form of GTP-bound Rap1a, which was then immunoprecipitated with glutathione resin. Rap1a activation levels were determined by Western blot using a Rap1a primary antibody (R&D Systems, catalog no.: AF3767).

### Quantitative PCR

RNA was isolated from hepatocytes or liver tissue using TRIzol (Invitrogen), and complementary DNA was synthesized from 1 μg of RNA using a cDNA synthesis kit (Invitrogen). Real-time PCR was performed using a 7500 Real-Time PCR system and SYBR Green reagents (Applied Biosystems). Specific primer sets used were as follows*: Pcsk9* forward: GAGACCCAGAGGCTACAGATT, *Pcsk9* reverse: AATGTACTCCACATGGGGCAA; *Rplp0* (36B4 -housekeeping) forward: GCTCCAAGCAGATGCAGCA, *Rplp0* (36B4 -housekeeping) reverse: CCGGATGTGAGGCAGCAG.

### Statistical analysis

All results are presented as mean ± SEM. *P* values were calculated using the Student’s *t*-test for normally distributed data and the Mann-Whitney rank sum test for non-normally distributed data. For experiments with more than two groups, *P* values were calculated using one-way ANOVA for normally distributed data and the Kruskal-Wallis by rank test for non-normally distributed data.

## Results

### Geranylgeranyl isoprenoid restoration suppresses statin-induced PCSK9 independent of a change in *Pcsk9* mRNA and lowers LDL-C

**Fig. 1:**
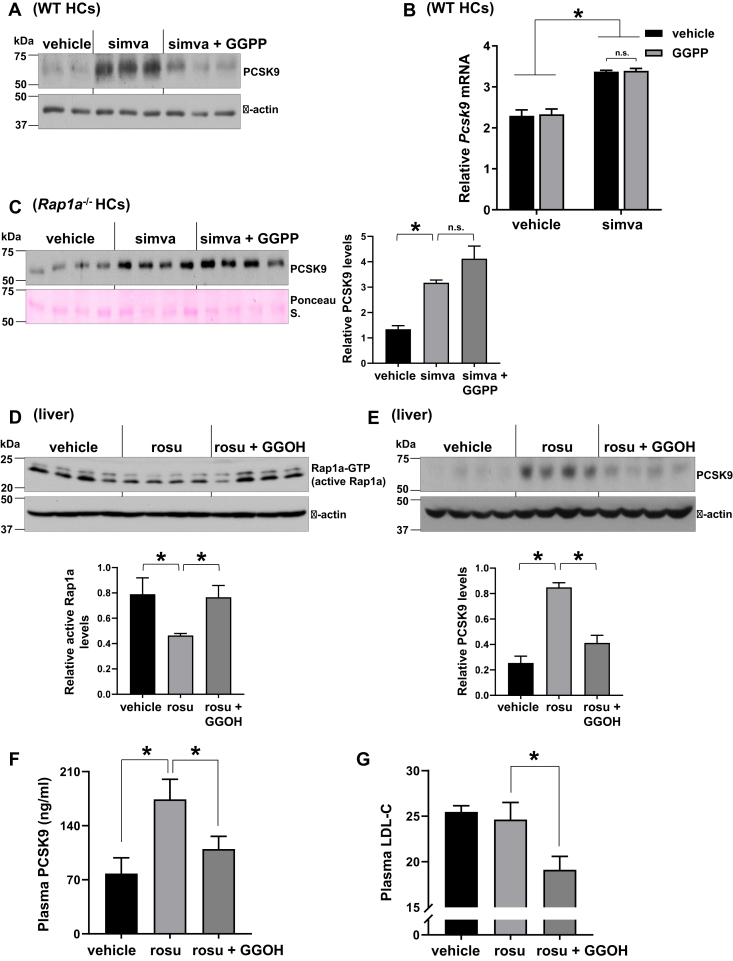
Geranylgeranyl isoprenoid restoration suppresses statin-induced PCSK9 independent of a change in *Pcsk9* mRNA and lowers LDL-C. A and B: Primary WT hepatocytes (WT HCs) were treated with vehicle, 10 μM simvastatin (simva), or simvastatin and 10 μM GGPP together for 32 h. PCSK9 protein (A) and *Pcsk9* mRNA (B) levels were measured (*n* = 2–4, mean ± SEM, ∗*P* < 0.05, n.s., nonsignificant). C: Same as in (A) except that *Rap1a*^−/−^ HCs were used and secreted PCSK9 was measured. Densitometric quantification of the immunoblot data is shown in the bar graph (*n* = 4, mean ± SEM, ∗*P* < 0.05, n.s., nonsignificant). D–G: Hepatic active Rap1a (D), liver PCSK9 protein (E), plasma PCSK9 (F), and plasma LDL-C (G) levels in high-fat diet-fed WT C57BL/6J male mice that were treated with vehicle, 10 mg/kg rosuvastatin (rosu), or rosu + GGOH (50 mg/kg) daily for 3 weeks. Densitometric quantification of the immunoblot data is shown in the bar graphs (*n* = 4–5, mean ± SEM, ∗*P* < 0.05).

Inhibition of HMG-CoA reductase by statins leads to reduced production of farnesyl pyrophosphate and GGPP isoprenoids. Because GGPP is required for Rap1a activation, and Rap1a is an important regulator of PCSK9 (36), we reasoned that a decrease in intracellular GGPP levels might be involved in statin-mediated PCSK9 induction. To test this, we restored intracellular GGPP in statin-treated hepatocytes by incubating them with exogenous GGPP, which we showed previously to bring back Rap1a′s prenylation and plasma membrane localization to control cells (37). Consistent with restoration of Rap1a′s membrane localization, GGPP treatment significantly lowered PCSK9 protein in statin-treated hepatocytes (Fig. 1A). GGPP treatment did not inhibit simvastatin-induced mRNA expression of *Pcsk9* (Fig. 1B) or other SREBP2 target genes, suggesting that activation of SREBP2 pathway upon statin treatment was not altered by GGPP restoration (37). Importantly, the ability of GGPP to lower statin-induced PCSK9 levels was abolished in Rap1a-deficient hepatocytes (Fig. 1C), supporting the idea that inhibition of Rap1a geranylgeranylation is one of the major mechanisms by which statins increase PCSK9 protein. We next investigated the functional importance in vivo and treated DIO WT mice with 10 mg/kg/day rosuvastatin alone or in combination with 50 mg/kg/day GGOH by daily gavage for 3 weeks. GGOH is an intermediate product in the mevalonate pathway and acts as a precursor to GGPP (44). Upon GGOH add back, hepatic Rap1a activity was restored and statin-induced hepatic and plasma PCSK9 levels were lowered (Fig. 1D–F). As WT mice are resistant to statins’ LDL-C-lowering effect due in part to strong induction of PCSK9 by statins (45), rosuvastatin alone had no effect on plasma LDL-C (Fig. 1G). However, when PCSK9 levels were lowered upon GGPP restoration, plasma LDL-C was decreased in statin-treated mice (Fig. 1G), without a change in body weight or food intake (not shown). These results support the hypothesis that inhibition of the mevalonate pathway by statins increases circulating PCSK9 in part via lowering intracellular GGPP levels, which is independent of the SREBP2-mediated *Pcsk9* mRNA induction.

### GGTase-I inhibition and prenylation-deficient Rap1a mimic statins and induce PCSK9 protein under basal conditions

**Fig. 2:**
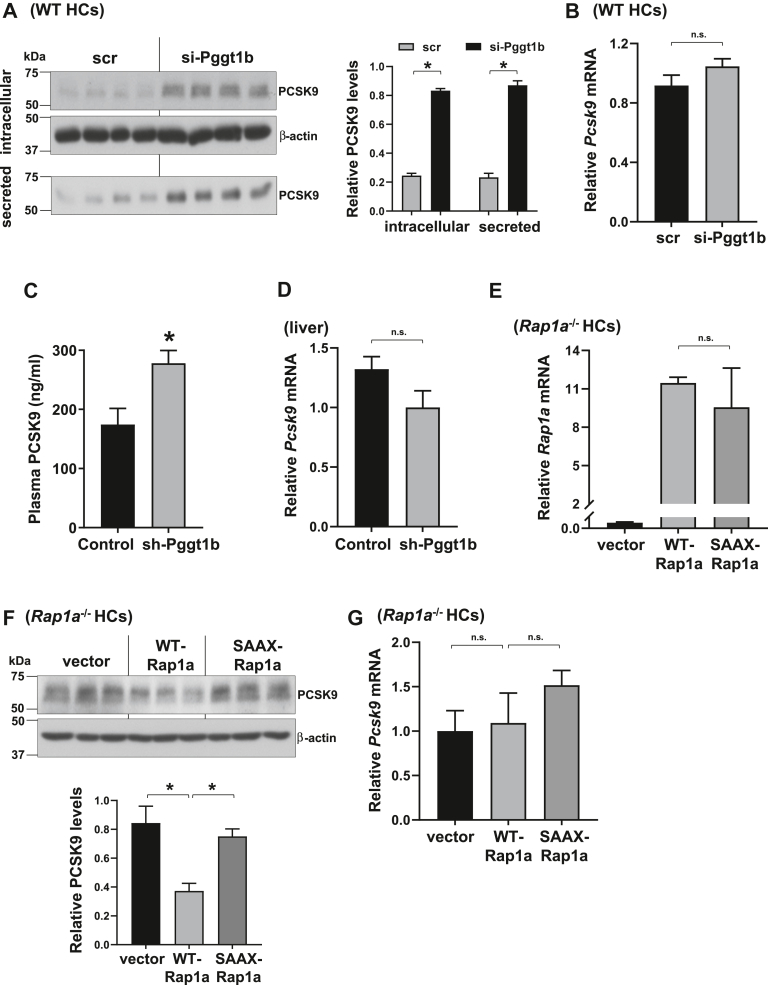
GGTase-I silencing and prenylation-deficient Rap1a increase PCSK9. A and B: intracellular and secreted PCSK9 protein (A) or *Pcsk9* mRNA (B) from scrambled (scr) or si-Pggt1b-treated primary WT hepatocytes (WT HCs). Densitometric quantification of the immunoblot data is shown in the bar graph (*n* = 4, mean ± SEM, ∗*P* < 0.05, n.s., nonsignificant). C and D: Plasma PCSK9 (C) and liver *Pcsk9* mRNA levels (D) from high-fat diet-fed WT C57BL/6J male mice treated with an AAV8-H1 construct encoding sh-Pggt1b or empty vector (control) (*n* = 7–8, mean ± SEM, ∗*P* < 0.05, n.s., nonsignificant). E–G: *Rap1a*^−/−^ HCs were transfected with empty plasmid (vector) or plasmids encoding WT or prenylation-deficient Rap1a (SAAX-Rap1a), and *Rap1a* mRNA (E), intracellular PCSK9 (F), and *Pcsk9* mRNA levels were assayed (G). Densitometric quantification of the immunoblot data is shown in the bar graph (*n* = 3, mean ± SEM, ∗*P* < 0.05, n.s., nonsignificant).

We next silenced GGTase-I, which catalyzes the covalent attachment of GGPP to the cysteine residue in the C-terminal CAAX motif of Rap1a to form a stable thioether bond (46, 47, 48). Similar to statin treatment or Rap1a inhibition, silencing *Pggt1b*, the gene encoding the GGTase-I enzyme, in hepatocytes increased both intracellular and secreted PCSK9 without affecting *Pcsk9* mRNA (Fig. 2A, B). In agreement with the in vitro data, silencing Pggt1b in the liver by treatment of WT mice with the hepatocyte-specific AAV8-H1-shPggt1b increased plasma PCSK9 and had no effect on hepatic *Pcsk9* mRNA levels (Fig. 2C, D). We then asked whether the prenylation-defective mutant form of Rap1a mimics statin treatment or GGTase-I deficiency and induces PCSK9. We transfected Rap1a-deficient hepatocytes with an empty plasmid or equal amounts of plasmids encoding the WT- or prenylation-deficient mutant form of Rap1a where the cysteine in the CAAX motif is replaced with serine to generate the nonprenylated Rap1a-SAAX mutant (Fig. 2E) (41). As expected, reconstituting WT Rap1a lowered PCSK9 protein as compared with empty plasmid-treated Rap1a-deficient cells, whereas Rap1a-SAAX mutant-treated cells expressed higher levels of PCSK9 protein without a change in *Pcsk9* mRNA (Fig. 2F, G). These data suggest the idea that inhibition of Rap1a prenylation induces PCSK9, which could contribute to the statin-PCSK9 link.

### Rap1a activation via 8-pCPT treatment suppresses basal and statin-induced PCSK9

**Fig. 3:**
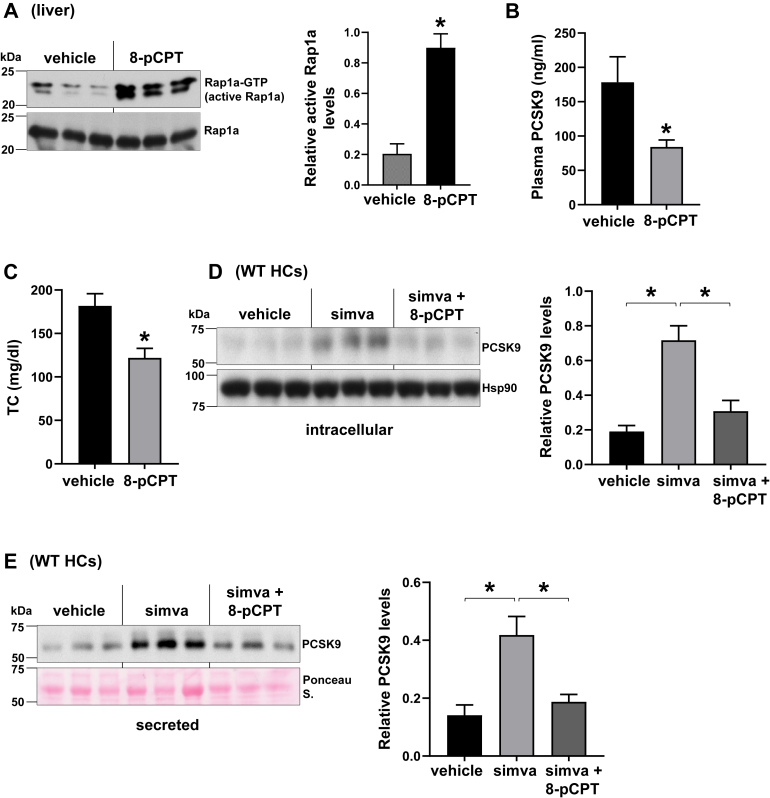
Rap1a activation via 8-pCPT treatment suppresses basal and statin-induced PCSK9. A–C: WT C57BL/6J male mice that were fed with high-fat diet were treated with 1.5 mg/kg/day with 8-pCPT for 2 weeks. Hepatic GTP-Rap1a (active Rap1a) (A), plasma PCSK9 (B), and plasma total cholesterol (TC) (C) were measured. Densitometric quantification of the immunoblot data is shown in the bar graph (*n* = 3–4, mean ± SEM, ∗*P* < 0.05). D and E: Intracellular (D) and secreted PCSK9 (E) levels were assayed in primary WT hepatocytes (WT HCs) treated with vehicle, 10 μm simvastatin (simva), or simvastatin and 20 μm 8-pCPT together for 48 h. Densitometric quantification of the immunoblot data is shown in the bar graphs (*n* = 3, mean ± SEM, ∗*P* < 0.05).

Because our previous work showed that overexpression of a constitutively active Rap1a lowers plasma PCSK9 (36), we asked whether activating Rap1a pharmacologically could have similar beneficial effects. The Epac activator, 8-pCPT, is a cAMP analog, where the 2′-*O*-methyl and 8-chlorophenylthio groups confer specific Epac binding and robustly activate Rap1a, and our previous work revealed that 8-pCPT treatment lowers PCSK9 secretion in isolated hepatocytes (36, 49). We next tested whether 8-pCPT can lower circulating PCSK9 in vivo. Based on the literature and our in vitro potency data, we treated high-fat diet-fed mice with 1.5 mg/kg body weight i.p. 8-pCPT daily for 2 weeks. The mice showed no signs of toxicity, and there were no differences in body weight or food intake by 8-pCPT (not shown). Rap1a activity in the liver was increased as evidenced by higher GTP-bound Rap1a levels (Fig. 3A), and as predicted by our hypothesis, 8-pCPT treatment lowered both plasma PCSK9 and total cholesterol levels (Fig. 3B, C). We then asked whether forced Rap1a activation via 8-pCPT treatment can overcome the statin-mediated Rap1a inhibition and lower PCSK9. We found that 8-pCPT pretreatment of isolated hepatocytes lowered simvastatin-induced intracellular and secreted PCSK9 protein (Fig. 3D, E). Collectively, these data provide additional support for the hypothesis that statins increase PCSK9 in part by inhibiting the GGPP-Rap1a pathway in hepatocytes.

### Rap1a silencing increases PCSK9 via inducing the RhoA-ROCK pathway

**Fig. 4:**
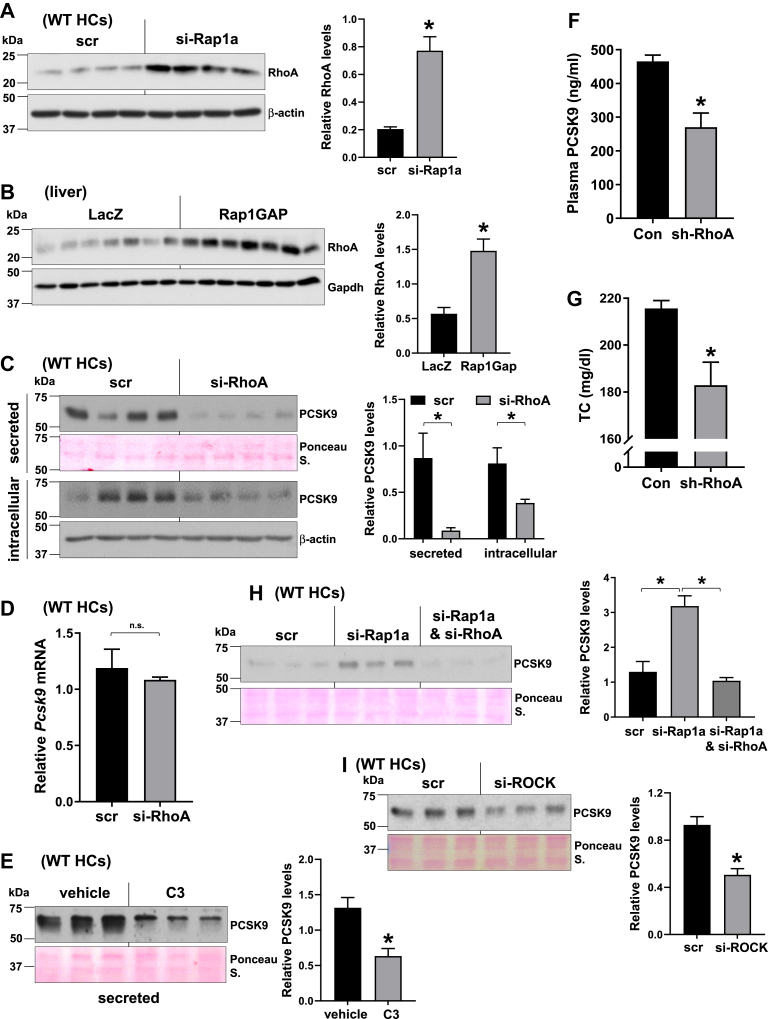
Rap1a silencing increases PCSK9 via inducing the RhoA-ROCK pathway. A and B: RhoA levels were assayed in primary WT hepatocytes (WT HCs) treated with scrambled (scr) versus si-Rap1a (A) or in adeno-LacZ versus adeno-Rap1GAP-expressing mice liver (B). Densitometric quantification of the immunoblot data is shown in the bar graphs (*n* = 4–7, mean ± SEM, ∗*P* < 0.05). C and D: Scrambled (scr) or si-RhoA-treated mouse HCs were assayed for secreted and intracellular PCSK9 (C) or *Pcsk9* mRNA (D). Densitometric quantification of the immunoblot data is shown in the bar graphs (*n* = 4, mean ± SEM, ∗*P* < 0.05). E: Mouse HCs were treated with vehicle or 1 μg/ml C3 transferase Rho inhibitor (C3), and secreted PCSK9 levels were assayed. Densitometric quantification of the immunoblot data is shown in the bar graphs (*n* = 3, mean ± SEM, ∗*P* < 0.05). F and G: Plasma PCSK9 (F) and plasma total cholesterol (TC) levels (G) were measured from AAV8-sh-RhoA or control AAV (Con)-treated mice fed with a high-cholesterol diet (*n* = 3–4/group, mean ± SEM, ∗*P* < 0.05). H and I: (H) Scramble (scr), si-Rap1a alone, or si-Rap1a + si-RhoA-treated primary HCs were assayed for secreted PCSK9. I: Same as in (H), except that scr- and si-ROCK-treated cells were used. Densitometric quantification of the immunoblot data is shown in the bar graphs (*n* = 3, mean ± SEM, ∗*P* < 0.05).

RhoA is a ubiquitously expressed small G protein, which gets activated by GTP loading (50). Although geranylgeranylation of RhoA is important for its plasma membrane localization, statin administration or inhibition of GGTase-I, which prenylates RhoA, paradoxically increases the protein expression and activity of RhoA in many cell types, including the hepatocytes (51, 52, 53, 54, 55). Similar to statin treatment, inhibition of Rap1a has been shown to induce RhoA and increase its active-GTP-bound form in endothelial cells (56, 57). In light of the proposed role of RhoA in LDLR regulation (55), we hypothesized that RhoA inhibition could be a key downstream event by which Rap1a regulates PCSK9 and LDLR. Supporting this idea, we first showed that Rap1a silencing in isolated hepatocytes increased RhoA levels (Fig. 4A). Correspondingly, we found that liver adeno-Rap1GAP overexpression, which inhibits Rap1a activity, increased RhoA protein, suggesting that Rap1a **→** ↓RhoA axis exists in liver (Fig. 4B). To test the importance of RhoA in PCSK9 and LDLR regulation, we silenced it in isolated hepatocytes. Similar to Rap1a activation, we observed that both intracellular and secreted PCSK9 were decreased upon si-RhoA treatment, without an effect on the *Pcsk9* mRNA (Fig. 4C, D). In addition, RhoA inhibition using the RhoA-specific inhibitor, C3, also lowered PCSK9 in hepatocytes (Fig. 4E). In order to assess the in vivo role of RhoA, we analyzed plasma and liver samples from control versus RhoA-silenced livers using hepatocyte-specific AAV8-H1-shRhoA (38) and found that liver RhoA silencing decreased plasma PCSK9 and plasma cholesterol levels (Fig. 4F, G). Next, we asked whether the increase in PCSK9 upon Rap1a inhibition is dependent on RhoA. To test this, we silenced RhoA in si-Rap1a-treated hepatocytes to decrease RhoA protein to a level similar to that in control cells. The data showed that the effect of Rap1a silencing on PCSK9 induction was abrogated when RhoA was also silenced (Fig. 4H), which suggest that the dominant pathway by which Rap1a inhibition induces PCSK9 is via increasing RhoA. We also tested whether the well-known RhoA effector, ROCK, participates in PCSK9 regulation and found that the ability of RhoA silencing to decrease intracellular and secreted PCSK9 was mimicked by silencing ROCK (Fig. 4I).

### Inhibition of RhoA-ROCK pathway enhances lysosomal-mediated degradation of PCSK9 and lowers statin-induced PCSK9 levels

**Fig. 5:**
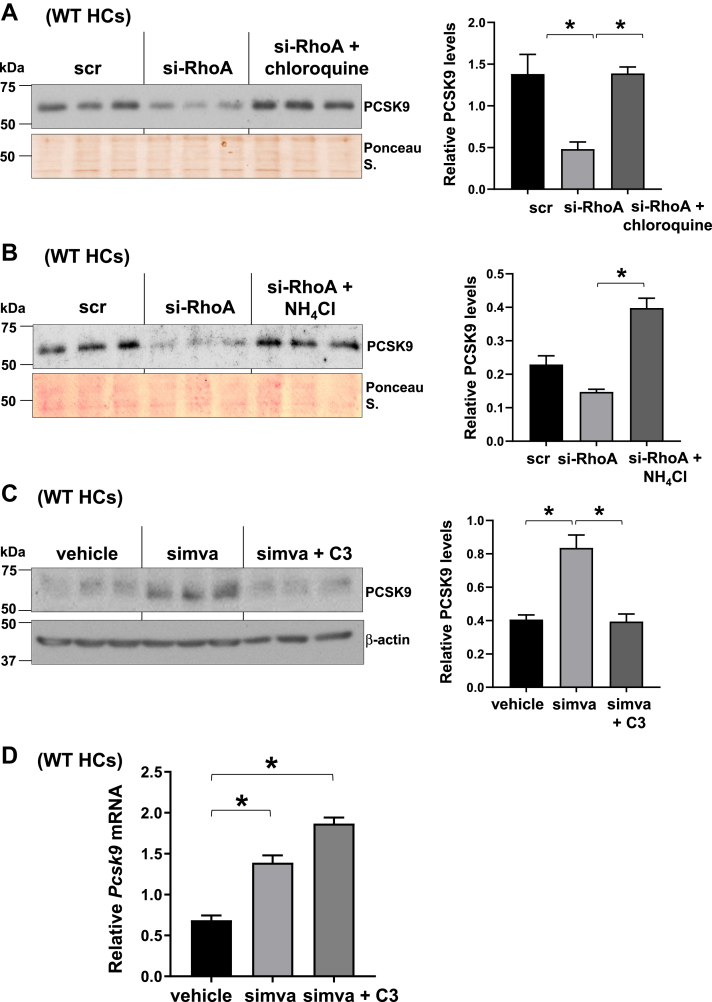
Inhibition of RhoA-ROCK pathway enhances lysosomal-mediated degradation of PCSK9 and lowers statin-induced PCSK9 levels. A and B: Secreted PCSK9 was assayed from scramble (scr) or si-RhoA-expressing primary WT hepatocytes (WT HCs) that were treated with chloroquine (A) or ammonium chloride (B). Densitometric quantification of the immunoblot data is shown in the bar graphs (*n* = 3, mean ± SEM, ∗*P* < 0.05). C and D: Intracellular PCSK9 protein (C) and *Pcsk9* mRNA (D) levels from primary HCs treated with vehicle, 10 μM simvastatin (simva), and simva + 1 μg/ml C3 for 32 h. Densitometric quantification of the immunoblot data is shown in the bar graphs (*n* = 3, mean ± SEM, ∗*P* < 0.05).

Previous studies revealed that PCSK9 gets degraded in the lysosomes, and our published work showed that activation of Rap1a increased the lysosomal degradation of PCSK9 (36, 58, 59). Correspondingly, we observed that disruption of lysosomal function via chloroquine or ammonium chloride treatment prevented the decrease in PCSK9 protein conferred by RhoA silencing (Fig. 5A, B), suggesting that RhoA inhibition, similar to Rap1a activation, enhances the lysosomal degradation of PCSK9. Finally, we found that RhoA silencing decreased statin-induced PCSK9 (Fig. 5C) without an effect on *Pcsk9* mRNA (Fig. 5D), which recapitulates GGPP restoration and Rap1a activator results. Altogether, these data suggest the existence of a pathway where activation of Rap1a suppresses RhoA and ROCK activity, which results in lower PCSK9 and contributes to LDL-C regulation.

## Discussion

**Fig. 6:**
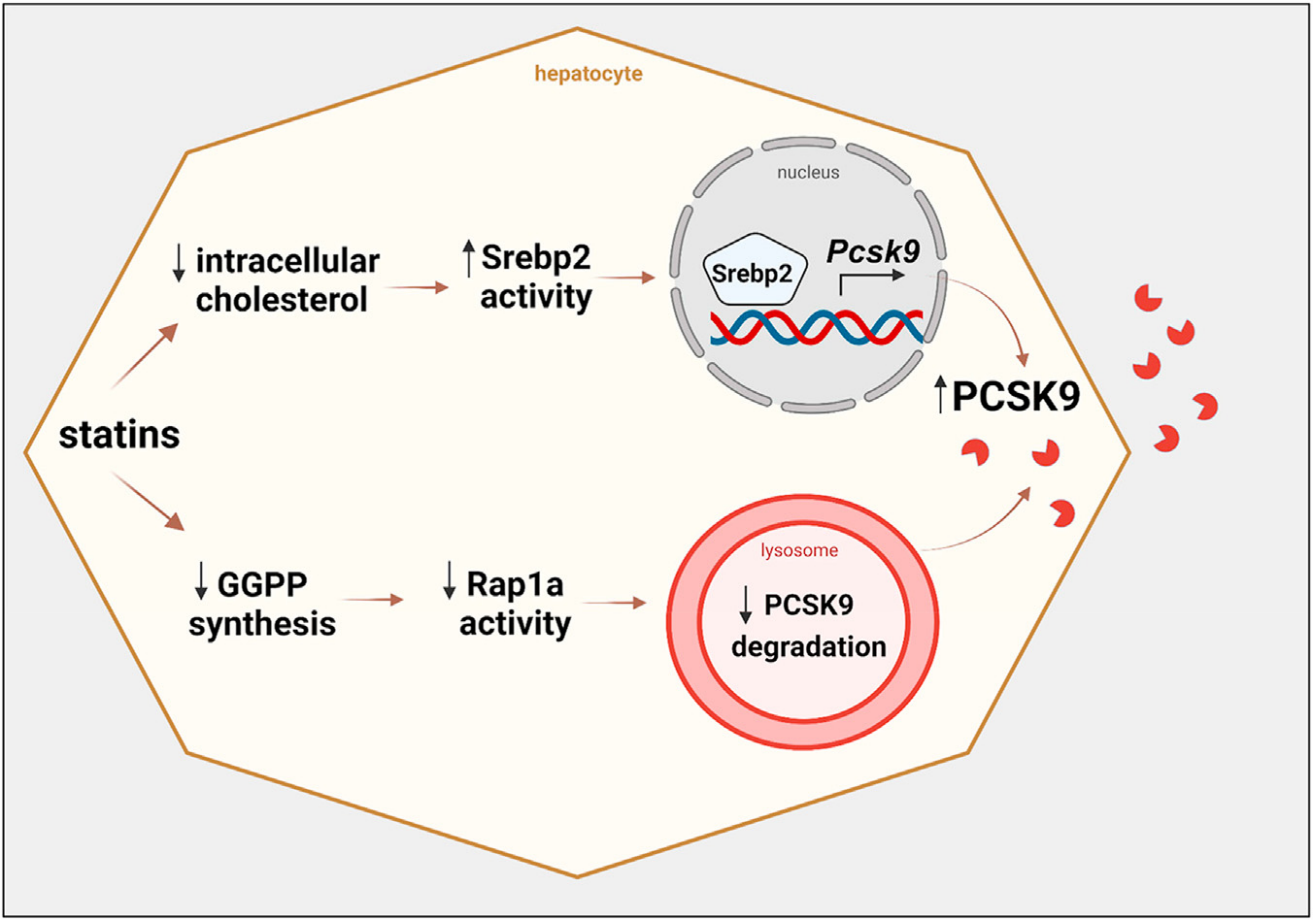
Proposed PCSK9 regulation by statins. Statins increase PCSK9 through increasing its mRNA via SREBP2 and inhibiting its degradation by decreasing the GGPP synthesis and Rap1a activity.

One mechanism by which statins paradoxically increase plasma PCSK9 levels is via inducing the SREBP2 pathway, which regulate PCSK9 at the mRNA level. Here, we report an additional mechanism that involves inhibition of GGPP synthesis and Rap1a activity, which upregulates PCSK9 post transcriptionally via the RhoA-ROCK pathway (Fig. 6). The data suggest that the same Rap1a-RhoA-ROCK pathway also regulates basal PCSK9 levels and can therapeutically be targeted via treatment with a Rap1a activator. In this regard, previous work has shown that the small-molecule activator of Rap1, 8-pCPT, protects against ischemia-reperfusion injury-induced renal failure and asthmatic airway inflammation (60, 61).

PCSK9 inhibitors are now used in the clinics and are well tolerated. Interestingly, genetic variants in *PCSK9* that mimic the effect of PCSK9 inhibitors are associated with increased risk for type 2 diabetes (T2D) development, raising the possibility that long-term PCSK9 inhibition may result in new-onset T2D (62, 63, 64, 65). Although this view has been challenged by many trials using anti-PCSK9 antibodies (66, 67), a recent meta-analysis of randomized clinical trials of PCSK9 inhibitors reported a modest increase in plasma glucose and HbA1C with anti-PCSK9 antibodies (68). In this regard, it is tempting to speculate that activating Rap1a has the benefit of improving T2D as we recently reported that Rap1a activation improves glucose tolerance in mouse models of T2D (37).

Our data suggest that Rap1a regulates PCSK9 via its downstream effectors, RhoA and ROCK, and previous work revealed that treatment of mice with a ROCK inhibitor lowered plasma cholesterol (69). Interestingly, a recent study reported that statins increased RhoA activity in human lymphoblastoid cells, which inversely correlated with their cell surface LDLR protein (55). Moreover, the greater statin-induced RhoA levels were associated with reduced LDL-C-lowering effect of statins in individuals from whom the cell lines were derived (55). In this regard, we postulate that our results provide a plausible explanation for the link between RhoA-ROCK and LDL-C metabolism and suggest that RhoA-ROCK-mediated PCSK9 regulation may be the key underlying mechanism of this effect.

In summary, the work presented here further describes an essential role for Rap1a prenylation and activity in the regulation of plasma PCSK9 and LDL-C. Furthermore, we provide evidence that GGPP supplementation and Rap1a activators can overcome the statin-mediated PCSK9 induction and increase their efficacy. Our results may suggest the future use of combination therapy of Rap1a activators or GGPP add back with lower doses of statins, which would increase statins’ tolerability and decrease side effects without compromising efficacy.

## Data availability

This study includes no data deposited in external repositories. All data are contained within the article.

## Conflict of interest

L. O. is an Editorial Board Member for Diabetes and was not involved in the editorial review or the decision to publish this article. All other authors declare that they have no conflicts of interest with the contents of this article.
